# Polymorphism of Genes and Their Impact on Beef Quality

**DOI:** 10.3390/cimb45060302

**Published:** 2023-05-30

**Authors:** Piotr Kostusiak, Jan Slósarz, Marcin Gołębiewski, Grzegorz Grodkowski, Kamila Puppel

**Affiliations:** Institute of Animal Science, Warsaw University of Life Sciences, Ciszewskiego 8, 02-786 Warsaw, Poland; piotr_kostusiak@sggw.edu.pl (P.K.); jan_slosarz@sggw.edu.pl (J.S.); marcin_golebiewski@sggw.edu.pl (M.G.); grzegorz_grodkowski@sggw.edu.pl (G.G.)

**Keywords:** cattle, beef, myostatin, thyroglobulin, calpain, calpastatin, SNP

## Abstract

The single-nucleotide polymorphism (SNP) form of genes is a valuable source of information regarding their suitability for use as specific markers of desirable traits in beef cattle breeding. For several decades, breeding work focused on improving production efficiency through optimizing the feed conversion ratio and improving daily gains and meat quality. Many research teams previously undertook research work on single-nucleotide polymorphism in myostatin (MSTN), thyroglobulin (TG), calpain (CAPN), and calpastatin (CAST) proteins. The literature review focuses on the most frequently addressed issues concerning these genes in beef cattle production and points to a number of relevant studies on the genes’ polymorphic forms. The four genes presented are worth considering during breeding work as a set of genes that can positively influence productivity and production quality.

## 1. Introduction

Population growth and the enrichment of many countries are increasing the demand for quality products [1]. One of these is beef, which is an important part of many people’s diets. A number of research teams previously undertook work to study the determinants of beef quality and looked for a way to implement the results of their research. This paper undertakes the task of reviewing the current state of knowledge about four candidate genes and the determination of their impact on beef quality. The genes highlighted in this work are myostatin (MSTN), thyroglobulin 5 (TG5), u-calpain (CAPN1), and calpastatin (CAST). Beef has long been the third most consumed meat after poultry and pork [2], with this trend due to its high price and, thus, higher consumer demand for quality [3]. In developed countries, it probably previously reached its peak per capita consumption, and for ethical reasons and due to environmental concerns, its consumption is now slightly decreasing [4]. Customers in highly developed countries prefer meat with a lower fat content [5,6]; this contradicts their taste choices in blind tests, in which they stated that they preferred the taste of beef with a higher fat content [7]. Beef with a higher intramuscular fat (IMF) content is also more nutritious [8]. Polymorphic forms of TG5 gene can increase intramuscular fat content by 6.5% [9] and can be used to further improve of cattle performance. This state of affairs, therefore, is a challenge for breeders, who must meet the demand of customers whose actual preferences seem to contradict the choices they make. Null mutation in MSTN results in 20–25% lower muscle mass in the Belgian Blue breed [10], which makes a significant difference in terms of productivity. To increase meat tenderness, work on CAPN1 and CAST, which improve meat quality, should be carried out at the same time [11]. Genomic selection is extremely important due to its effectiveness in the process of improving meat quality. Juiciness, color, tenderness, and water-holding capacity are important elements of beef quality [12]. Meat production worldwide exceeded 337 million tons in 2020 [13], and this growth was accompanied by an increasing interest in higher quality products [14,15]. It is, therefore, necessary to conduct work on candidate genes, which can be an important tool in shaping breeding programs and will expand our knowledge about important issues, such as production efficiency and meat quality. The literature was selected based on keywords related to the topic of the paper in several bibliographic databases of the Warsaw University of Life Sciences, including Web of Science and Scopus.

## 2. Myostatin

Myostatin (MSTN), also known as GDF8 (growth and differentiation factor 8), is one of the most important regulators of skeletal muscle development [16]. It is a highly conserved gene that can be identified in many mammal livestock species [10]. This gene plays a crucial role in muscle size development [17]. Huang et al. reported that a lack of MSTN activity resulted in the overgrowth of skeletal muscles—this issue is called the double muscling (DM) trait [18]. The double muscling phenotype is described using a number of symbols: DM or N, DM or dm, D or n, C or N, A or a, and mh or + [19]. It is highly desired among cattle producers due to its positive impact on the meat content of carcasses. MSTN can generate both a significantly higher proportion of skeletal muscle on the carcasses of slaughtered animals and the expression of adipose tissue through inhibiting or promoting adipogenesis [20]. Muscles can be as much as 20–25% larger than those of individuals without the mutation [10], while a decrease in the proportion of organ mass [21,22] and a reduced proportion of fat on the carcass are also associated traits [23].

All members of the TGF -β (transforming growth factor -β) family are characterized by three distinct domains: the N-terminal signal domain, the C-terminal mature peptide, and the propeptide domain [10]. In cattle, these trait were mapped at chromosome 2 [24]. Other similar characteristics, such as the hydrophobic core of amino acids near the N-terminal signal domain, cysteine residues in the C terminal region, and the conserved RSRR proteolytic processing signal at the C-terminus, indicate that MSTN is a member of this family [25]. However, there is a difference that distinguishes MSTN from the rest of this family—the shorter nucleotide sequences at the C-terminus.

MSTN expression might be identified in various tissues. It can be identified in mammary glands, lymphocytes, spleen, and the cardiomyocytes in heart tissue, and it has an important role in skeletal muscle development [26]. It consists of three exons and two introns. The exons code for a 375 amino-acid (aa) latent protein, which later becomes biologically active through post-translational modification. Through forming disulfide bonds, the polypeptide undergoes intracellular homodimerization [27,28]. Two forms are produced: the N-terminal propeptide region and the C-terminal mature region. These forms initiate intracellular signaling cascades due to their ability to bind and activate the type II activin receptor located on the cell surface (ActRIIB and ActRIIA). Subsequently, the autophosphorylation process of ActRIIB l leads to the recruitment and activation of the low-affinity activin type I receptors ALK-4 or ALK-5. Through phosphorylating the transcription factors Smad2 and Smad3 with activated type I receptor kinase, it is possible for them to interact with Smad4 (co-Smad) and translocate to the nucleus in order to activate the transcription of the target gene [29]. The activated MSTN receptor is able to inhibit protein kinase B, which determines muscle protein synthesis and cell proliferation. The process of increasing the size of muscle fibers is called muscle fiber hypertrophy (or hypertrophy for short) and is strongly regulated through Protein kinase B (Akt). The formation of mature skeletal muscles is the result of myogenic differentiation. The high proliferation muscle precursors formed during embryogenesis differentiate into myoblasts. Myostatin determines the regulation of pre-natal muscle development processes through affecting myoblast proliferation, muscle precursors, and differentiation [30]. MSTN also affects the regulation of the marker that initiates the proliferation of the muscle precursor Pax3 in limb muscles. MSTN is additionally responsible for the increased expression of p21, which stops the proliferation of myoblasts that express myoblast determination protein 1 (MyoD), which is an important regulator of MSTN expression during myogenesis [20].

The phenomenon of muscle hypertrophy was previously identified in many mammalian species. However, muscle hypertrophy is not an accurate term because, in many cases, muscle growth is due to pre-natal hyperplasia [31]. The literature indicates that in species such as cattle, horses, sheep, and goats, changes in MSTN gene expression mainly cause hyperplasia, while hypertrophy is observed in mice [32]. Thus, the term is used casually. Muscles with larger surface areas increase their size significantly, while deeper muscles tend to decrease in size compared to normal muscles. When raising beef cattle, muscle size is very important, as it affects the conformation of the carcass and the proportions of the valuable elements that determine profits from the sale of the animal. Reduced body weights and body fats were observed in obese rats using sActRIIB or a polyclonal antibody to MSTN [33].

MSTN is an important component in myogenesis. However, this action is not its only function that affects production yield. Some sources report that it plays an important role in adipogenesis. MSTN can inhibit either adipogenesis in preadipocytes or can promote it in pluripotent stem cells. The deletion or inhibition of MSTN might improve muscle mass and reduce fat mass [34,35,36]. To determine the effect of MSTN on adipogenesis, white and brown adipocytes should be distinguished. White adipocytes are responsible for storing energy in large lipid droplets, while brown adipocytes contain much more numerous small droplets, which are used in non-shivering thermogenesis [37]. Studies report that MSTN can not only inhibit the adipogenesis of white adipocytes but also of brown adipocytes. This process involves Smad3-mediated β-catenin stabilization and TGF-β/Smad3 signaling [38]. Moreover, it was previously proven that under certain conditions of adipogenesis, mouse embryonic fibroblasts can differentiate into brown fat cells. MSTN-deficient primary mouse embryonic fibroblasts show differentiation into brown adipocytes with increased lipid metabolism [39]. MSTN inhibition can lead to a reduction in subcutaneous body fat in mammals. Transgenic mice whose propeptide cDNA sequence had suppressed MSTN showed reduced subcutaneous, epididymal, and retroperitoneal adipose tissue compared to normal animals [40]. McPherron and Lee [41] concluded that myostatin inhibition may be more effective at limiting adipose tissue gain than reducing it when soluble MSTN receptors from the extracellular domain of type IIB activin receptors were used in mice and induced through a high-fat diet. Reduced body weight and body fat were observed in obese rats using sActRIIB or a polyclonal antibody to MSTN. McPherron and Lee observed that white adipose tissue was converted to brown [41].

There are many reports on the effects of the various allelic variants through which breeding work was carried out to improve both slaughter yield and the quality of the meat itself, as well as reports of inactive MSTN on traits related to growth rate and carcass conformation [20,42,43]. The result of these works is the consolidation of the DM trait in the population, which is a desirable trait because of its beneficial effects on production efficiency and raising the quality of meat—a characteristic that is desired by consumers [44]. These animals have a lower proportion of bone and fat in the carcass, significantly higher proportions of muscle, a lower proportion of connective tissue, and improved meat tenderness. Many researchers focused their work on studying this phenomenon for specific breeds (Table 1).

One of the best-known cattle breeds with the DM trait is the Belgian Blue [49]. Many years of selection for this trait resulted in it being accentuated to an unprecedented level. According to a study by Grobet et al. [24], this breed has an 11-bp deletion (g.821–831 del11) in the open reading frame. There is the loss of three amino acids (275–277) and a shift in the reading frame after aa 274, resulting in a stop codon after aa 287. 

The differences between DM and normal cattle can be seen in many aspects of the slaughter performance. The carcass’ lower fat and bone content, the lower collagen content of the muscles, and the significant increase in the size of some muscles relative to normal cattle all account for the value of this trait in beef cattle production conditions [50]. In Belgian Blue cattle, the semitendinosus muscle can be 1.6 times larger than in normal animals [51]. There are also significant changes in subcutaneous and intramuscular fat content. It is worth separating these two types of fat due to the consumption value of the meat. European customers prefer lean beef; thus, these changes are particularly welcome. The situation is, however, different in the markets of many countries where reduced intramuscular fat reduces the steaks’ attractiveness [52,53,54]. 

Myostatin plays a key role in the processes of adipogenesis and myogenesis. The deletion and inhibition of MSTN contributes mainly to an increase in size of individual skeletal muscles and a reduction in the proportion of fat in the carcass. These are important traits from the breeder’s point of view; thus, they are often used in crossbreeding to improve slaughter performance.

The DM phenotype is characterized by significant muscle hypertrophy relative to normal individuals. There is significant muscle prominence in the hindquarter and anterior quadrant areas, with clearly defined individual muscle parts separated by grooves. Breeding work led to the consolidation of the DM trait, which improves production results. It was only after some time that research began on the impact of this trait on animal health. Arthur et al. pointed out health problems in such animals, stating that lower fertility and lower calf viability were observed [55,56]. Dystocia is another problem found in DM cattle [57]. The well-muscled hindquarters and the effect of hyperplasia on calves prior to birth result in a higher frequency of dystocia. Belgian Blue cattle are the best example of this—almost every parturition ends with a cesarean section. It also turns out that these animals are more likely to become ill, as evidenced by an increased frequency of disease entities involving the respiratory, urinary, digestive, motor, and many other systems. For breeders, the effect on reproduction is also important. Animals with this trait are characterized by higher birth weights, which makes calving more difficult [54]. In addition, DM cattle have increased proportions of glycolytic muscle fibers, which are characterized by a susceptibility to fatigue; thus, these animals show a lower resistance to physical exertion and a faster onset of metabolic acidosis [58]. 

## 3. Thyroglobulin

Thyroglobulin (TG) is the main protein of the thyroid gland and makes up to 75% of the gland’s protein [59]. The thyroglobulin gene is considered to be a candidate gene that affects the ability to accumulate intramuscular fat; thus, it is important for breeders and further breeding work. Thyroglobulin production takes place in the thyroid gland’s follicular cells, and is secreted from the endoplasmic reticulum into a site where it undergoes iodination (incorporation of iodine into the tyrosine residues of thyroglobulin). It is stored inside thyroid follicles [60]. As a glycoprotein homodimer, it is a substrate in the production of the thyroid’s hormones and a carrier of triiodothyronine (T3) and tetraiodothyronine (T4) (called thyroxine). The influence of thyroid hormones is important for the regulation of metabolism and its effects on the growth, differentiation, and homeostasis of fat cell composition [61]. Thus, these are important hormones that affect the development of fat cells. Hormone release occurs due to the stimulation of the thyroid cells by the thyrotropic hormone (TSH). Further activity by the released hormones stimulates the hepatic processes of gluconeogenesis and lipogenesis, as well as the occurrence of glycogenolysis [62].

The TG5 gene is one of the longest genes in mammals. In cattle, it is located in the centromere region of the fourteenth chromosome, and consists of 37 exons. It is made up of two allelic variants, i.e., TG5C and TG5T, and three genotypes, i.e., TG5CT, TG5DW, and TG5T [63,64]. This gene affects the accumulation of body fat and is used for animal selection based on a single nucleotide polymorphism (SNP) located in the 5′ untranslated region of this gene [65].

Intramuscular fat content is an important factor in determining the quality of beef. This trait positively correlated with the juiciness and palatability of meat, and improves its flavor, tenderness, and nutritional value. Meat rich in intramuscular fat is characterized by a higher content of fat-soluble vitamins and unsaturated fatty acids [66]. This characteristic is referred to as meat marbling, and influences consumers’ interest during the purchase [67]. Most of the intramuscular fat is located between bundles of muscle fibers in the perimysium connective tissue [68]. 

Intramuscular fat deposition can be influenced by factors such as sex, weaning age, age and weight at slaughter, nutrition, and environmental factors. However, the trend in the quantitative change in intramuscular fat that is under the influence of the mentioned factors is related to breed [69]. Genetic potential largely determines the final marbling score (MS) [69].

In studies by Rincker et al. [70] and Casas et al., the TG5 SNP had no clear effect on beef marbling [70,71]. This result could be due to the rearing period being too short (<250 days) or other factors. Wood et al., in their meta-analysis based on 11 papers, indicated that there was a positive association between the polymorphic forms of TG5 and the degree of meat marbling [72]. A significant relationship between beef quality and TG5 for Charolaise and Angus cattle was also determined by Van Eenennaam et al. [73]. They indicated there was a significantly higher IMF content for TT genotypes compared to CC. Moreover, in the work of Barendse et al. on a sample containing 1750 cattle, it was indicated that TG5 can be used as an effective tool to improve marbling [9].

Park et al. [69], on the basis of papers written by Albrecht et al. [74] and Irie et al. [75], determined that the average IMF content in the longissimus dorsi (LD) muscle in the Japanese Wagyu breed was 36.5%; for the Korean Hanwoo breed, it was 13.7% [76,77,78,79], while for the Angus breed, it was 7.1% [80,81,82]. For the Hereford crossbreed, the figure was 6.9%. In research by Dubovskova et al., the presence of TT homozygote at 5% was determined in beef characterized by good marbling, and this also had the best results in terms of IMF content [83].

## 4. The Calpain–Calpastatin System

In the case of CAST and CAPN1, the influence on meat tenderness variability is more than 40% [84]. Thus, they are an extremely important element in the beef production process and have a very strong impact on the quality of the final product. Work carried out on beef tenderness is very important for improving meat quality. Out of the group of genes on which research has been conducted for decades, most of the work focused on calpains (CAPN) and calpastatins (CAST), which are the CAPN inhibitors. From 1993 to 2021, there were at least 175 English-language papers related to the topic [85]. This is a clear signal that work should be conducted to analyze the factors affecting meat quality, in particular tenderness (associated both with CAST [86] and CAPN1 [87,88]), which is the most important determinant of the customers’ willingness to buy. The variability in genes in the calpain–calpastatin system depends on the breed of cattle; therefore, the use of SNPs as genetic markers for animal selection to improve genetic progress is a promising direction [89,90]. Smith et al. indicated that meat tenderness is 46% determined by genetic factors and 54% determined by environmental factors [91].

Calpains are considered a candidate for being responsible for the meat tenderization process, alongside Takahashi’s calcium tenderization theory [92]. Many authors argue that the calpains that are dependent on the presence of calcium ions are responsible for this process. There is the consideration that these calpains may be responsible by virtue of their access to substrates, their ability to hydrolyze proteins, and because they are also found within the cells of muscle tissue. Evidence for their actions are indicated via the reduction in proteolysis under the influence of calcium ion chelators [93], as well as zinc chloride [94]. The main components of the calpain system are µ-calpain (CAPN1), m-calpain (CAPN2), calpain 3 (originally named p94, CAPN3), and their specific inhibitor, i.e., calpastatin (CAST), which blocks their activity [95]. Calpains are found in the cytoplasm of all vertebrate cells [96]. They are named after the calcium ion concentration required for their activation: µ-calpain requires 3–50 µM of calcium and m-calpain 0.4–0.8 mM to reach half of its maximum activity. In live animals, calcium concentration in the muscles is 0.2 µM [97], and only after slaughter does the calcium ion concentration rise to 100 µM [98], thus allowing µ-calpain activation. CAPN1 is considered to be the most important element in the maturation of meat due to its early activation stage, which occurs after slaughter. CAPN2 is activated later, when the calcium ion concentration increases further. CAPN2 is, therefore, important in the later stages of meat maturation. For CAPN3, no significant effect on the post-mortem proteolysis of meat was found [86], though some results may be promising [99,100,101]. The activity of µ-calpain and calpastatin fall sharply in the first few days after slaughter [102], which correlates with an increase in meat tenderness [103]. Boehm et al., Koohmaraie, and Pringle et al., confirmed that calpain plays a major role in this process [93,104,105]. CAPN1 4751 and CAPN1 316 were addressed by research teams in several studies (Table 2), and are largely responsible for meat tenderness in *Bos taurus* and *Bos indicus* cattle, as well as in their crosses. In the case of CAPN4751, a significant effect on tenderness was confirmed by Morris et al. [106], while in the case of CAPN316, the cutting force was decreased by about 20% [107] in *Bos taurus* crosses, which can be used as a meat quality predictor. For the CAPN1 530 marker, no significant effect on meat tenderness was observed in any breed.

Along with µ-Calpain, calpastatins are endogenous calcium-dependent proteinases that are responsible for mediating the proteolysis of myofibrillar proteins during meat aging processes [120]. CAPN1 is responsible for the proteolysis of cytoskeleton proteins and intermediate filaments. Endogenous proteases called calpains and their inhibitor (calpastatin) are thought to be responsible for initiating the degradation of myofibrillar proteins after slaughter [95].

The degradation of cytoskeletal and myofibrillar proteins largely influences changes in muscle cell integrity, which determines the degree to which the meat is tender and shapes organoleptic parameters. This process is shaped through the right aging conditions, such as temperature, time, and type of aging (dry or wet), which affect changes in meat pH over time. The activation of endogenous proteolytic enzymes is necessary to trigger these processes. One of the most important enzymes is µ-calpain, which digests desmin structures and is encoded by the CAPN1 gene. Barendse et al. indicated a strong epistatic effect between the CAST and CAPN1 genes, which occurs in most breeds [121]. The study observed that substituting alanine for glycine in CAPN1: c.947G > C had the greatest effect on meat tenderness in the Angus and Belmont Red cattle breeds. 

Changes in consumer needs and eating habits require breeders to make breeding progress and continually improve product quality. A number of studies carried out on calpain also included calpastatin [122], which, as its inhibitor, plays an important role in the maturation of meat [115]. There are several forms of calpastatin, such as CAST, CAST1, CAST2, CAST3, and CAST4 [123]. Calpastatin is also dependent on calcium ions. In a study by Malheiros et al., which was conducted on *Bos Indicus*, significantly higher expressions of the CAST2 isoform were observed for hard meat and very hard meat compared to medium–hard meat [124]. For the CAST and CAST1 isoforms, no significant differences were observed between the experimental groups. In a study by Muroya et al., variations in expression were observed depending on the type of muscle [125]. Such results indicate that there are variable expressions of CAST isoforms in different muscles (Table 3), which may result in different calpain inhibition and, thus affect meat maturation differently.

In a study by Allais et al., which was conducted on three beef cattle breeds (Charolaise, Limousine, and Blonde d’Aquitaine), differences were found between the CAST SNP results for each breed [126]. For the Blonde d’Aquitaine breed, an increase in required cutting power and a decrease in tenderness were observed for the GA haplotype on the CAST-2 and Cast-3 markers. Casas et al. demonstrated the additive effect of the CAST-2 G allele in the GPE cycle7 group [111], which confirmed the findings of Allais et al. and, at the same time, indicated the positive effect of CAST TT on meat tenderness [126]. Similar conclusions were reached by Johnston and Graser in the case of required cutting strength for the CRC1 population, using Angus, Hereford, and Murray Grey breeds as examples [127]. The G allele was found to have a reduced effect in Charolaise x Angus, Brahman, and Hereford populations [73]. The important role of CAST and the relationship between CAST and CAPN1 in the regulation of beef tenderness was also confirmed by Tait et al. and Lee et al. [112,128].

## 5. Conclusions

The MSTN SNP, also known as DM, is associated with a mutation in the myostatin gene that affects muscle hypertrophy relative to normal individuals, and can be used to identify the double muscle phenotype in the further selection of individuals. The identification and isolation of the gene makes it possible to distinguish between heterozygous and homozygous individuals, which provides a significant advantage in achieving genetic progress in breeding and, thus, achieving more efficient production. DM is either hyperplasia, which increases the number of muscle fibers pre-natally, or hypertrophy, which manifests itself as an increase in muscle fiber diameter post-natally. The trait is widespread in some European cattle populations, particularly Belgian Blue cattle.

The thyroglobulin gene (TG5) is an important determinant of the degree of meat marbling. Intramuscular fat content is an important element from the consumer’s point of view, and enables the identification of individuals characterized by higher (TT genotype) or lower (CC genotype) proportions of fat in the muscle, which, depending on the market, can be desirable or undesirable. The TG5 SNP will allow breeding directions to be adapted to the needs of consumers in the market.

Calpain and calpastatin are the main determinants of the degree of tenderness in beef. Thus, they are an extremely important factor in the meat maturation process, which is the last stage of production; therefore, any loss in quality at this stage has the greatest consequences. CAST and CAPN1 SNP, through their influence, significantly enhance organoleptic qualities, and breeding work that utilizes them can significantly improve beef quality. It makes sense to identify those individuals that are characterized by the best meat tenderness, as thos strategy encourages customers to buy beef products.

Single nucleotide polymorphisms are a kind of signpost for beef cattle breeders. They are very helpful in the process of breeding progress and allow for more rational decision-making in the selection of individuals. They are also important in scientific and research work. However, it should be remembered that realizing the genetic potential of animals requires the highest possible level of welfare and optimal environmental conditions. Many environmental factors can negatively affect animal weight gain and meat quality. Among the most important factors that we can point to is heat stress, which can cause changes in the color of meat, reduce daily gains [129], and negatively affect reproduction [130]. It is important to provide proper rearing conditions to take advantage of the genetic potential of the animals. Careful analysis of SNPs and the study of their effects on animals is an important research direction, and it is important to carry out further work to learn as much as possible about the operation of such critically important genes in breeding.

## Figures and Tables

**Table 1:** MSTN gene polymorphism in cattle breeds.

	SNP		
Reference	Breed	Position	Mutation
[24]	Belgian Blue	c.821	Del11
[10]	Blonde d’Aquitaine	c.821	Del11
[45]		g.3811	T > G
[46]	Charolaise	c.610	C > T
[46]	Limousine	c.821	Del11
[47]		c.610	C > T
[48]		g.433	C > A
[47]	Marchigiana	g.874	G > T
[46]	Piedmontese	c.938	G > A

**Table 2:** CAPN gene polymorphism in cattle breeds.

Reference	Breed	Muscle	CAPN SNP
[108]	Angus, Charolaise, Brahman, and Nguni	Longissimus thoracis et lumborum	CAPN1 184^+^, CAPN1 187^+^, CAPN1 4751^+^, and CAPN2 780^+^
[109]	Charolaise, Limousine, and Retinta	Longissimus dorsi	CAPN1^+^
[106]	Jersey–Limousine cross, Angus, and Hereford cross	Longissimus dorsi	CAPN1: c.947C > G^+^
[110]	Piedmontese–Angus cross and Jersey-Limousine cross	Longissimus thoracis	38 SNPs^+^
[111]	Angus, Red Angus, Beefmaster, Brangus, Hereford, Bonsmara, Romosinuano, Brahman, Limousine, Charolaise, Gelbvieh, and Simmental	No data	CAPN1^+^
[107]	Brangus, Beefmaster, Bonsmara, Brahman, Romosinuano, Hereford, and Angus	Longissimus	CAPN1 316^+^, CAPN1 4753^+^, and CAPN1 530^+^
[112]	Hanwoo	Longissimus lumborum	CAPN1:c.1589G > A^+^, CAPN1:c.658C > T^+^, CAPN1:c.948G > C^+^, and CAPN1:c.580A > G^+^
[113]	*B. taurus*, *B. indicus,* and crosses	Longissimus dorsi	CAPN1 316^+^ and CAPN1 4751^+^
[114]	Brahman	Longissimus dorsi	CAPN316^+^ and CAPN4751^+^
[115]	Nellore	Longissimus dorsi	CAPN1 316^+^, CAPN1 4751^+^, CAPN1 530^+^, and CAPN1 4753^+^
[116]	Nellore	Longissimus dorsi	CAPN1 4751^−^
[117]	Nellore	Longissimus dorsi	CAPN1 4751+
[118]	Turkish Grey	Longissimus dorsi	CAPN1 316^+^ and CAPN1 4751^+^
[119]	Parda de Montaña and Pirenaica	Longissimus thoracis	CAPN1 316^−^, CAPN1 530^−^, and CAPN1 4751^−^

No association with meat tenderness, (−); association with meat tenderness, (+).

**Table 3:** CAST gene polymorphism in cattle breeds.

Reference	Breed	Muscle	CAST SNP
[108]	Angus, Charolaise, Brahman, and Nguni	Longissimus thoracis et lumborum	CAST 736^+^ and CAST 763^+^
[109]	Charolaise, Limousine, and Retinta	Longissimus dorsi	CAST^+^
[106]	Jersey–Limousine cross, Angus–Hereford, and other crosses	Longissimus dorsi	CAST: c.2959A > G^+^
[111]	Angus, Red Angus, Beefmaster, Brangus, Hereford, Bonsmara, Romosinuano, Brahman Limousine, Charolaise, Gelbvieh, and Simmental	No data	CAST^+^
[112]	Hanwoo	Longissimus lumborum	CAST:c.182A > G^+^, CAST:c.1985G > C^+^, and CAST:c.1526T > C^+^
[113]	*B. taurus*, *B. indicus*, and crosses	Longissimus dorsi	CAST-T1^−^
[114]	Brahman	Longissimus dorsi	CAST^+^
[115]	Nellore	Longissimus dorsi	UOGCAST^+^ and WSUCAST^+^
[117]	Nellore	Longissimus dorsi	UOGCAST^+^
[118]	Turkish Grey	Longissimus dorsi	UOGCAST^+^
[119]	Parda de Montaña and Pirenaica	Longissimus thoracis	CAST1^+^, CAST2^+^, CAST3^−^, CAST4^+^, and CAST5^−^

No association with meat tenderness, (−); association with meat tenderness, (+).

## Data Availability

All data generated or analyzed during the study are included in this published article. The datasets used and/or analyzed in the current study are available from the corresponding author on reasonable request.
